# Essential Oils and Bioproducts for Flea Control: A Critical Review

**DOI:** 10.3390/insects16121276

**Published:** 2025-12-16

**Authors:** Diefrey Ribeiro Campos, Ingrid Lins Raquel de Jesus, Fabio Barbour Scott, Thais Ribeiro Correia, Yara Peluso Cid

**Affiliations:** 1 Department of Animal Parasitology, Veterinary Institute, Federal Rural University of Rio de Janeiro, Seropédica 23897-000, Brazil; raquellingrid@gmail.com (I.L.R.d.J.); scott.fabio@gmail.com (F.B.S.); thaisrca@gmail.com (T.R.C.); 2 Department of Pharmaceutical Sciences, Institute of Biological and Health Sciences, Federal Rural University of Rio de Janeiro, Seropédica 23897-000, Brazil; yarapcid@gmail.com

**Keywords:** *Ctenocephalides*, Siphonaptera, *Pulex*, *Xenopsylla*, volatile oils, green chemistry

## Abstract

Fleas have major veterinary and public health relevance by causing dermatopathies and transmitting zoonotic agents. The limitations and environmental impact of synthetic insecticides have driven the search for safer and more sustainable tick control alternatives, such as essential oils (EOs) and their bioactive compounds. We conducted searches in the PubMed, Scopus and Google Scholar databases (May–August 2025), using predefined inclusion and exclusion criteria, resulting in 25 studies. The species investigated most was *Ctenocephalides felis*, followed by *Xenopsylla cheopis*. The most common botanical families were Lamiaceae, Cupressaceae, Rutaceae, and Lauraceae, comprising 48 plant species. For *C. felis*, the EOs of *Cinnamomum* spp., *Ocimum gratissimum*, *Syzygium aromaticum* and Piper aduncum showed the most pronounced insecticidal effects, as did their bioactive compounds cinnamaldehyde, eugenol, and dillapiole, respectively. For *X. cheopis*, the EOs of *Cinnamomum verum*, *Calocedrus decurrens*, and *Salvia rosmarinus* were the most effective. Essential oils from different plant species also exhibited repellent activity against *C. felis*, *Pulex irritans* and *Diamanus montanus*. In summary, essential oils and their bioactive compounds have promising potential for the control of fleas of medical and veterinary importance, representing sustainable alternatives with reduced environmental impacts.

## Simple Summary

Fleas are common parasites of pets and wildlife, and they can transmit diseases that also affect humans. The control of these insects usually depends on synthetic chemical products, which can cause environmental contamination and lead to resistance. In recent years, scientists have been exploring natural alternatives, such as essential oils extracted from plants. In this review, we analyzed 25 scientific studies that tested essential oils and their active compounds against different flea species. The cat flea was the most frequently studied, followed by the rat flea. Essential oils and bioactive compounds showed strong insecticidal effects, while some also acted as repellents. These findings suggest that essential oils and their bioactive compounds may serve as effective and eco-friendly options for controlling fleas that impact both animal and human health.

## 1. Background

### 1.1. Fleas

Fleas are ectoparasitic insects belonging to the order Siphonaptera, which is composed exclusively of hematophagous species, at least during the adult stage of their life cycle. Currently, this order includes approximately 2200 described species, distributed across 19 recognized families. Most species parasitize mammals, with only a small fraction, approximately 5%, being associated with birds [1]. Among these families, Pulicidae and Tungidae stand out due to the presence of species with high medical, veterinary, and public health relevance, attributed to their broad geographic distribution, vector capacity, and clinical impact on hosts [2,3].

Within the Pulicidae, *Ctenocephalides felis* (Bouché, 1835) is currently the most prevalent and widely studied flea species worldwide, due to its broad distribution, and close association with dogs and cats [2,3,4]. *Ctenocephalides canis* (Curtis, 1926) shares similar morphology with *C. felis* but has more restricted distribution, being primarily found in temperate regions [5,6]. Other important species within this family include *Xenopsylla cheopis* (Rothschild, 1903), the primary vector of bubonic plague and murine typhus [7,8], and *Pulex irritans* (Lineus, 1758), commonly known as the human flea, which can parasitize various mammals and is also associated with the transmission of pathogens in specific epidemiological contexts [9,10,11,12,13,14].

Although other flea species of medical and veterinary importance exist, this review focuses on the biology, significance and control strategies of *C. felis*, since it is the most extensively studied flea species on a global scale. Other species are discussed when relevant, particularly in relation to the application of essential oils or bioactive compounds for their control.

#### 1.1.1. Biology and Ecology

Fleas are holometabolous insects that undergo a four-stage life cycle consisting of egg, larva, pupa and adult [12,13]. Adults are obligate hematophagous ectoparasites that live primarily on the host, where they feed, mate and oviposit within 24–48 h after the first blood meal [14,15,16,17]. Eggs lack adhesion and readily fall into the environment, accumulating in resting sites such as bedding, carpets and cracks [12,18,19]. Under optimal conditions (20–30 °C; >50% RH), larvae hatch within 1–6 days and progress through three instars, feeding on organic debris and especially adult flea feces rich in partially digested blood [15,20,21]. After 5–11 days, mature larvae spin a silk cocoon incorporating environmental particles and pupation occurs for 5–14 days, although quiescent pupae may persist for weeks to months until stimulated by heat, CO_2_ or vibration [13,18,22,23,24]. The life cycle generally lasts 21–28 days, and in an infested environment, adults represent only a small fraction of the total flea population [13,18].

Fleas are cosmopolitan ectoparasites that infest numerous mammalian hosts and, to a lesser extent, birds [1]. *Ctenocephalides felis* is the most widely distributed species globally, parasitizing dogs, cats and various wild mammals, whereas *C. canis* has more restricted distribution [25,26,27,28,29]. Other medically important species include *P. irritans*, *X. cheopis*, a key vector of plague and murine typhus, and *T. penetrans*, a zoonotic burrowing flea [7,8,11,30,31,32,33]. Many fleas persist in sylvatic cycles where wildlife reservoirs maintain parasite populations and facilitate spillover into domestic animals, especially in regions where urban areas overlap with natural habitats [34,35,36]. Environmental factors, particularly temperature and humidity, strongly influence flea development and population dynamics, and ongoing climate change is expected to expand flea distributions, prolong periods of activity and increase the risk of flea-borne diseases [6,37,38,39,40].

#### 1.1.2. Medical and Veterinary Importance

*Ctenocephalides felis* and *C. canis* are the main flea species parasitizing companion animals worldwide, being responsible for a wide range of clinical manifestations [41]. The most significant condition associated with infestation by these species is flea allergy dermatitis (FAD), a hypersensitivity reaction to allergens present in flea saliva. FAD is considered one of the most common dermatological disorders in dogs and cats, and is characterized by intense pruritus, erythema, alopecia, excoriations, secondary pyoderma, and in chronic cases, lichenification and hyperpigmentation [42,43]. This condition significantly impacts the quality of life of affected animals and often requires ongoing multidisciplinary management [44].

In addition to direct clinical effects, these fleas play an important role in the transmission of infectious agents of both veterinary and zoonotic relevance. *C. felis* is a recognized vector of *Rickettsia felis*, the causative agent of flea-borne spotted fever in humans, as well as *Bartonella henselae*, the etiological agent of cat scratch disease, a zoonosis primarily transmitted through infected felines [45,46]. Both *C. felis* and *C. canis* serve as intermediate hosts of the cestode *Dipylidium caninum*, whose transmission occurs mainly through the accidental ingestion of infected fleas, especially posing a potential source of infection in children [47]. These fleas can also transmit *Acanthocheilonema reconditum*, a subcutaneous filarial nematode in dogs [48,49].

Other flea species, although less frequently found on pets, have notable medical significance. *Xenopsylla cheopis*, commonly referred to as the “oriental rat flea,” is historically recognized as the primary vector of bubonic plague, caused by *Yersinia pestis* [8,50], and is also involved in the transmission of *Rickettsia typhi*, the causative agent of murine typhus [51,52]. This species is typically associated with urban rodents and poses a potential threat in unsanitary conditions and densely populated areas. *Pulex irritans*, the “human flea,” has broad geographic distribution and low host specificity, having been reported on humans, pigs, dogs and other mammals. Its importance lies both in the discomfort caused by its bites and in its potential to transmit *Rickettsia* spp. and *Bartonella* spp. [30,53].

Finally, *T. penetrans*, known as the “chigoe flea” or “jigger,” has a unique biological cycle compared to other flea species. The fertilized female actively penetrates the host’s skin, typically in the plantar regions, where it enlarges and develops, causing intense local inflammation and pain, secondary infection, and in severe cases, deformities [54,55]. This species is endemic to tropical and subtropical areas and has a significant impact on both human and animal health, especially in communities with poor sanitary conditions [56].

#### 1.1.3. Control Strategies and Challenges

Flea control has undergone significant evolution over recent decades, in parallel with advances in the research and development of molecules offering greater efficacy, selectivity, and safety for both animals and the environment. Initially, broad-spectrum compounds such as organochlorines were used. These substances provided prolonged residual activity but were associated with high mammalian toxicity and environmental accumulation [27,57,58]. During the 1960s and 1970s, they were gradually replaced by organophosphates, such as chlorpyrifos [58,59,60], and carbamates, such as propoxur and carbaryl [61,62,63]. These acetylcholinesterase inhibitors were effective but had significant toxic potential for both mammals and ecosystems, so organophosphates are currently banned in several countries [64,65].

In the 1980s, pyrethroids including permethrin, cypermethrin and deltamethrin became widely adopted due to their rapid action and greater selectivity. Despite their initially good performance, these compounds suffered progressive reduction in efficacy due to the emergence of resistance in flea populations [66,67,68,69]. The 1990s marked a significant advance with the introduction of insect growth disruptors, such as lufenuron, which inhibits chitin synthesis [70,71,72]; methoprene; and pyriproxyfen, a juvenile hormone analog [73,74,75]. Although these compounds are ineffective against adult fleas, they play a fundamental role in controlling immature stages and interrupting the flea life cycle [76].

Also in the 1990s, neonicotinoids such as imidacloprid [77,78], and subsequently dinotefuran [77,79], were introduced. These compounds act as agonists of nicotinic acetylcholine receptors, causing neural hyperexcitation and parasite death [80]. They are characterized by having rapid action onset, a broad spectrum and favorable safety profile, especially in topical formulations. Around the same time, fipronil became widely used. Belonging to the phenylpyrazole class, fipronil acts as an antagonist of GABA-gated chloride channels and became popular due to its adulticidal efficacy [81,82,83]. Another important innovation was the introduction of selamectin, a macrocyclic lactone derived from avermectins. It is the only compound in this class with proven efficacy against fleas, offering monthly topical control through both endoparasiticidal and ectoparasiticidal activity [84,85].

In the early 2000s, spinosad, a compound of the spinosyn class, began to be used as a fast-acting oral alternative, especially for animals intolerant to topical treatments [86,87]. Beginning in 2014, the introduction of isoxazolines revolutionized systemic flea control. Compounds such as fluralaner [88], afoxolaner [89] sarolaner [90], lotilaner [91] and esafoxolaner [92] act on GABA and glutamate-gated chloride channels, providing potent, prolonged and highly selective adulticidal activity, with excellent safety profiles for dogs and cats [93].

Despite these therapeutic advances, flea control remains challenging due to the complexity of the flea life cycle. Most immature forms. including eggs, larvae and pupae, are found in the environment in protected locations such as carpets, crevices, fabrics and outdoor areas. These stages promote reinfestation even after effective treatment of the host [27,94,95]. Resistance is also an increasing concern, not only to older compounds such as pyrethroids, organophosphates and carbamates, but also to modern molecules. Populations of fleas with genotypes resistant to fipronil and neonicotinoids have already been identified [57,96,97,98]. Resistance mechanisms include mutations at binding sites, such as ion channels and nicotinic receptors, as well as upregulation of detoxification enzymes, including cytochrome P450s, esterases and glutathione S-transferases. Continuous use, underdosing and lack of chemical class rotation have contributed to the selection of resistant strains [98,99].

Besides therapeutic and genetic aspects, growing concern exists regarding the environmental impacts of veterinary ectoparasiticides [100]. Topical and systemic compounds can contaminate the environment through excretion, bathing or simple contact with surfaces, reaching aquatic and terrestrial ecosystems. These residues can affect non-target organisms such as aquatic invertebrates and beneficial arthropods, and they can persist in the environment [101,102,103]. The absence of specific environmental regulations for veterinary pharmaceuticals further complicates mitigation efforts [103].

In this context, effective and sustainable flea control requires an integrated approach that combines rational and rotational use of pharmaceuticals, appropriate environmental management practices, constant monitoring of therapeutic efficacy, resistance surveillance and the inclusion of natural alternatives with lower ecological impact, such as essential oils (EOs) and bioactive compounds. Adoption of this strategy, aligned with the One Health concept, is essential to ensure long-term and responsible control of these ectoparasitoses.

### 1.2. Essential Oils and Bioactive Compounds

Essential oils are concentrated natural plant products that contain a mixture of volatile constituents produced by the secondary metabolism of aromatic plants and other plant varieties [104]. Plants, as stationary organisms, have developed many chemicals (secondary metabolites) to protect themselves against predators (such as insects) and pathogens [105]. Their chemical composition provides a broad bioactive spectrum (antimicrobial, insecticide/acaricide, repellent, anti-inflammatory) and a long tradition of medicinal use [104].

Physicochemically, EOs are defined as complex mixtures of volatile, fat-soluble, and generally colorless compounds [104,106]. Their chemical composition may include hundreds of constituents, but the number, diversity and relative abundance of these compounds vary according to species, genotype, chemotype, plant organ, environmental conditions and extraction method, all of which directly influence their potency and spectrum of biological activity. The variability in EO content arises from both intrinsic and extrinsic factors. Intrinsic ones are governed by the plant’s genetic makeup [107], with genotype being the primary determinant of the concentration of each compound present [108]. Among extrinsic factors are conditions such as seasonality, water availability, and soil fertility [109]. Additionally, temperature, humidity, nutrient supply, light intensity, photoperiod and ecological interactions can modulate EOs’ composition [110]. Moreover, post-harvest factors, including storage conditions and whether plant material is used fresh or dried, further contribute to compositional differences [111].

A plant’s genotype corresponds to the set of genetic information that determines its biosynthetic potential, including the enzymatic pathways responsible for secondary metabolites’ formation. Genetic differences can result in qualitative (presence or absence of certain compounds) and quantitative (higher or lower concentrations) changes, directly influencing the chemical composition, and consequently the biological activity of the EOs [104,112]. On the other hand, the chemotype represents the observable phenotypic manifestation of this potential, characterized by the dominant chemical profile in the EO, defined by the major compounds. Different chemotypes can be found in morphologically identical individuals of the same species, each associated with distinct bioactivities [113,114]. Thus, while the genotype provides the genetic basis for the production of metabolites, the chemotype expresses the result of the interaction between genetics and the environment, being decisive for the standardization, efficacy and safety of the use of EOs in therapeutic contexts.

Like all organic compounds, EOs are composed of hydrocarbon molecules and can be classified as terpenes, alcohols, esters, aldehydes, ketones and phenols, oxygenated compounds, monoterpene alcohols, sesquiterpene alcohols, aldehydes, esters, lactones, coumarins, ethers and oxides [104]. However, the most important active compounds fall into two chemical groups: terpenoids (monoterpenoids and sesquiterpenoids) and phenylpropanoids [104,112].

The insecticidal activity of EOs is multifactorial, and precise elucidation of their biological mechanisms of action is challenging due to the diversity of their compounds [115]. Among the most common targets are those of a neurochemical/neurohormonal nature, such as inhibition of GABA (Gamma-amminobutyric acid) receptors, changes in the activity of acetylcholinesterases (AChEs), interference with glutamate-gated chloride channels (GluCls) and inhibition of octopamine and tyramine receptors (OARs/TARs) [116]. Other targets, such as antioxidant defensive enzymatic systems, dysregulation of insect development in different stages of the life cycle (insect growth disruptors—IGDs). Repellent and deterrent effects on insects have also been described [115,117,118]. Moreover, the effect of EOs on cuticular permeability plays an important role, either directly by displacing the lipids that form the cuticle and causing dehydration, and consequently insect death, or indirectly, by acting as penetration enhancers that improve transdermal drug delivery [117,118]. Terpenes, terpenoids, and phenylpropanoids have been shown to interact with AChE activity, with α-pinene, carvacrol, limonene, menthol, menthone, and 1,8-cineole being among the most effective. The phenylpropanoids eugenol and cinnamaldehyde have been reported to interact with OARs/TARs. The monoterpenoids carvacrol, pulegone, and thymol were identified as positive allosteric modulators of the insect GABA receptor. In addition, thymol and menthol have been shown to inhibit GluCls [117]. However, studies using specific receptors from flea species are still lacking.

The potential of EOs against insects of agricultural, veterinary and medical relevance is well established, and numerous reviews have been published on this topic [119,120], including their use for the control of veterinary ectoparasites [121]. The major body of research on EOs describes their activity against mosquitoes and ticks. In the past decade, several studies have reported pulicidal activity of EOs and isolated compounds. Nevertheless, there is a lack of reviews focused on EOs and fleas, both related to insecticidal activity or repellency, as well as the relationship between EO composition and activity. The aim of this review is to contextualize the insecticidal and repellent potential of EOs and their active constituents against fleas of medical and veterinary importance, summarizing the studies conducted to date while also highlighting current limitations and future perspectives in this field.

## 2. Methods

### 2.1. Article Search Strategy

We conducted a bibliographic search in the PubMed, Scopus and Google Scholar databases, covering the period from May to August 2025. The objective was to identify studies investigating the activity of essential oils and their major constituents against different flea species, with emphasis on the bioprospecting of novel compounds with potential flea-control activity. Specific combinations of search terms were employed, including: “essential oil” AND “Siphonaptera”; “essential oil” AND “fleas”; “isolated compounds” AND “Siphonaptera”; “isolated compounds” AND “fleas”; “essential oil compounds” AND “Siphonaptera”; “essential oil compounds” AND “fleas”; “bioactives” AND “Siphonaptera”; and “bioactives” AND “fleas”.

### 2.2. Article Selection Criteria

After removing duplicate articles, the eligibility of the remaining studies was assessed according to the predefined inclusion criteria for this review. Initially, the title and abstract were read as the first screening criterion. Studies that demonstrated suitable scope of the review were subjected to full reading to confirm eligibility.

Only original articles published in English were included, provided that their primary objective was related to evaluating the flea-control activity of EOs and their major constituents, defined as the ability to induce mortality or repellency in fleas. Studies addressing plant extracts, research focusing on ectoparasites other than fleas, or general reviews on the use of EOs against different parasites were excluded.

Due to the large number of results returned by certain search terms in Google Scholar, the initial screening was performed by reading article titles. The search was concluded after assessing three consecutive pages without relevant articles, ensuring a systematic management of publication volume without compromising the inclusion of pertinent studies. Articles meeting these criteria were considered eligible for inclusion in the review.

### 2.3. Data Extraction and Analysis Criteria

From the eligible articles, detailed information was extracted regarding study design, including: flea species investigated; essential oil or major constituent used; developmental stage; type of study (*in vitro* or *in vivo*); formulation development (when applicable, specifying bioactive concentration, formulation type, and excipients); assessed biological activity parameters, expressed as lethal concentration or repellent efficacy, where the former is defined as the ability to induce flea mortality and the latter as the capacity to repel fleas without necessarily causing immediate or subsequent death; supplementary studies (residual efficacy and knockdown effect); mortality mechanism; and mortality assessment criteria.

**Figure 1:**
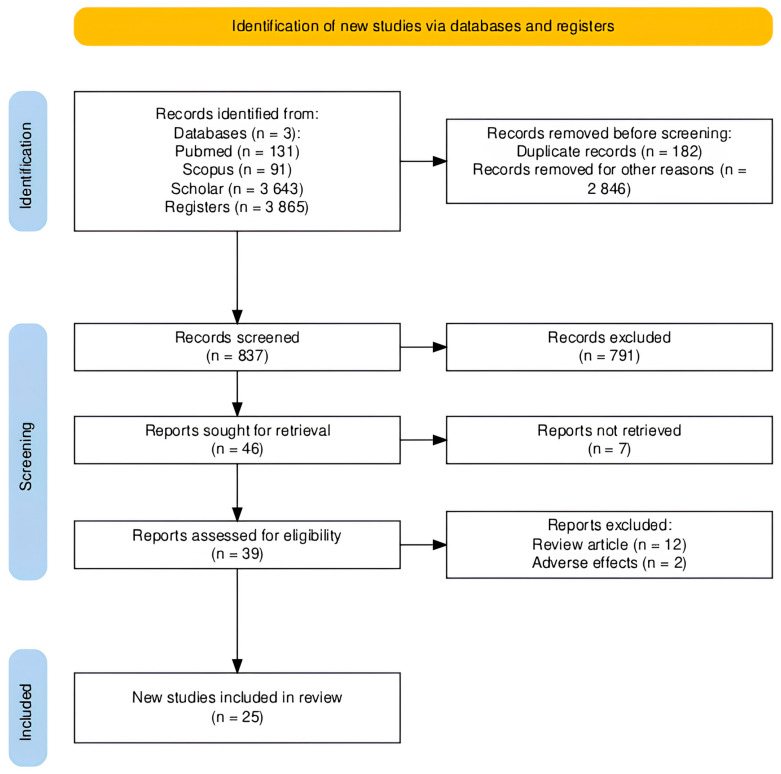
Flowchart of the identification, processing, and final selection of articles included in the review. Generated using PRISMA Flow Diagram.

Among the identified articles, only 25 met the predefined inclusion and exclusion criteria, and were grouped according to the biological activity studied, as previously mentioned. The complete process of article search, screening and selection for inclusion in the review is summarized in Figure 1, which provides an overview of the selection workflow, including the number of studies identified, duplicates removed, and articles deemed eligible, using a flow diagram generated with the PRISMA Flow Diagram web application (https://estech.shinyapps.io/prisma_flowdiagram/, accessed on 9 October 2025) in accordance with PRISMA guidelines.

## 3. Results

### 3.1. General Results

**Figure 2:**
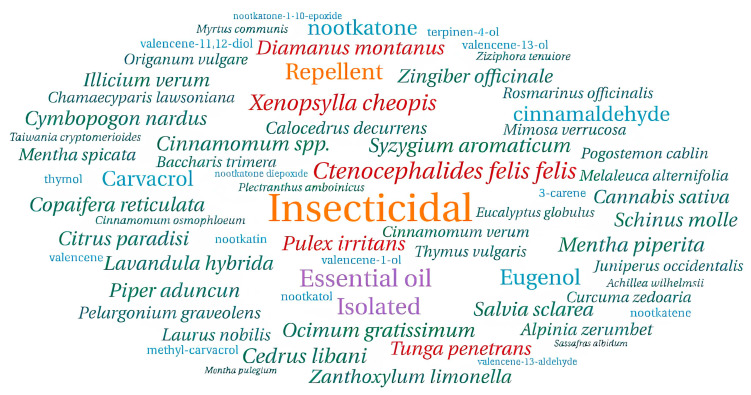
Word cloud representing the frequency of keywords extracted from the selected studies. The most prominent terms, including “*Ctenocephalides felis felis*”, “essential oil”, and “insecticide”, reflect the main research focus areas within the analyzed literature. Generated using WordArt.com, accessed on 9 October 2025.

A total of 25 studies met the inclusion criteria, reporting *in vitro* or *in vivo* biological activity of essential oils and/or their bioactive compounds against fleas, either through controlled laboratory assays or formulated preparations. Among these, 16 studies exclusively evaluated EOs, five assessed both EOs and their major bioactive compounds, and one investigated isolated bioactive compounds only. Furthermore, four studies focused on the development of formulations targeting fleas, of which two employed bioactive compounds as active ingredients and two utilized whole EOs, as illustrated in the word cloud summarizing frequent keywords (Figure 2).

The flea species most frequently investigated was *C. felis*, with 17 studies evaluating the activity of essential oils and/or their major constituents against one or more developmental stages. In second place, *X. cheopis* was addressed in five studies, while *T. penetrans*, *P. irritans*, and *Diamanus montanus* (Siphonaptera, Ceratophyllidae) were each represented by a single publication. The majority of studies (n = 20) focused on the insecticidal activity of EOs or bioactive compounds against different life stages, whereas three assessed repellent effects, and two evaluated both insecticidal and repellent activities.

The analysis of botanical diversity revealed that the species used belong to 16 botanical families, comprising a total of 48 species evaluated for their insecticidal and/or repellent activity against fleas. Among these botanical families, Lamiaceae had the highest number of representatives (12 species), followed by Cupressaceae (6 species), Rutaceae (5 species), and Lauraceae (5 species). The families Zingiberaceae and Myrtaceae included three and four species, respectively, while Asteraceae and Fabaceae comprised two species. The remaining families, Anacardiaceae, Cannabaceae, Geraniaceae, Pinaceae, Piperaceae, Poaceae, Schisandraceae, and Ericaceae, were each represented by a single species.

Regarding the use of isolated bioactive components from EOs against fleas, eight published studies were identified that evaluated the *in vitro* insecticidal or repellent activity of these compounds. All told, 19 bioactive components were assessed, including 11 sesquiterpenes (such as nootkatone, valencene, and α-cadinol, among others), five monoterpenes (carvacrol, thymol, terpinen-4-ol, among others), and three phenylpropanoids (eugenol, cinnamaldehyde and dillapiole).

Of the 25 studies analyzed, 22 evaluated the activity of EOs or their bioactive compounds through *in vitro* assays. In these experiments, flea mortality or repellency was determined based on direct contact of the insects with filter papers or glass surfaces previously treated with the tested compounds. In contrast, only two studies employed *in vivo* assays, both designed to evaluate the repellent effect of EOs against fleas, and only one study evaluated the *in vivo* insecticidal activity of an EO against fleas.

**Figure 3:**
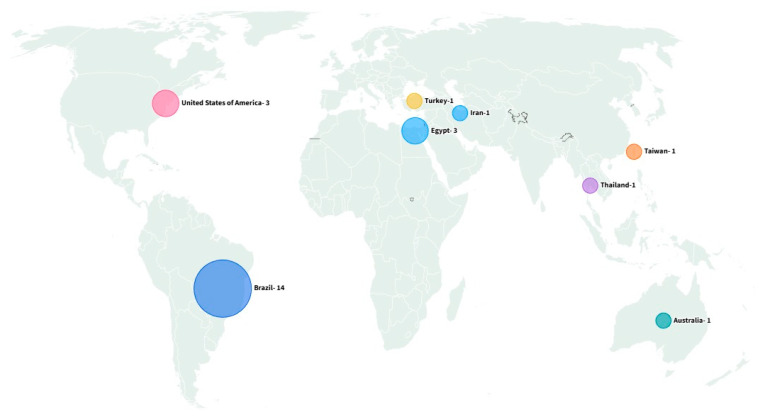
Global distribution of the studies selected for this review, according to country of origin. Flourish online platform (https://flourish.studio/, accessed on 9 October 2025).

Regarding the geographical distribution of publications, the highest number of studies were conducted in Brazil (n = 14), the main contributor to the literature on the use of essential oils and bioactive compounds for flea control. These data are visualized in Figure 3 as a geographical map. Other countries with published studies included Egypt (n = 3), the United States (n = 3), and Australia, Iran, Taiwan, Thailand, and Turkey, each with one publication (Figure 3). This distribution indicates that Brazilian researchers have contributed the largest number of studies, reflecting a growing national interest in the use of essential oils and bioactive compounds as alternative strategies for flea control.

**Figure 4:**
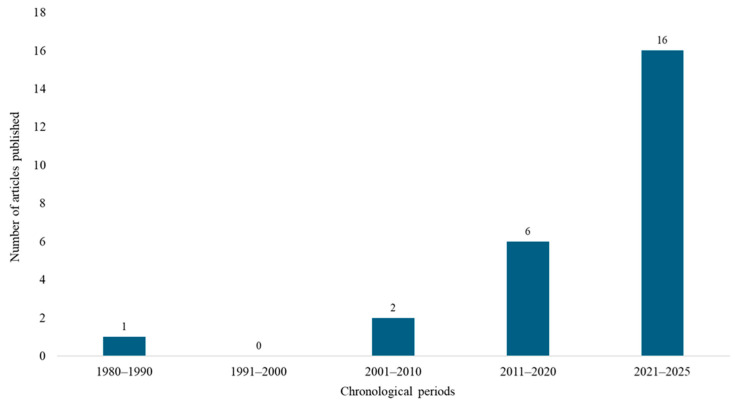
Temporal evolution distribution of scientific publications on the bioprospecting of essential oils and their major constituents for flea control over the decades.

In relation to the temporal distribution of publications, studies were first reported between 1980 and 1990 (n = 1), followed by a gap in the 1990s, with no publications identified in that decade. The number of studies gradually increased in the following years, with two articles published between 2001 and 2010, six between 2011 and 2020, and a marked rise to 16 publications from 2021 to 2025. This trend indicates noteworthy growth in research interest and productivity over the last decade, reflecting the increasing scientific and applied relevance of natural products for flea control (Figure 4).

### 3.2. Insecticidal Activity of Essential Oils

#### 3.2.1. Immature Stages

Studies evaluating the efficacy of EOs against immature stages of fleas were identified only for the species *C. felis.* Regarding the egg stage, 10 articles assessed the ovicidal activity of EOs from 17 plant species (Table 1). When comparing the LC_50_ values of these EOs, we observed a wide variation. The lowest LC_50_ values, corresponding to the highest ovicidal activity, were recorded for *Cinnamomum* spp., with LC_50_ values ranging from 90 to 150 µg·mL^−1^, and *Ocimum gratissimum*, ranging from 89.5 to 527.2 µg·mL^−1^. Conversely, the highest LC_50_ values were obtained for *Baccharis trimera* (15,647.2 µg·mL^−1^) and *Salvia sclarea* (19,510.0 µg·mL^−1^), indicating low efficacy against the egg stage. Some species, such as *Citrus paradisi*, *Copaifera reticulata*, and *Schinus molle*, showed no detectable biological activity under the evaluated conditions.

Larvicidal activity was evaluated in nine studies that investigated the essential oils of 16 plant species (Table 1). The lowest LC_50_ values were recorded for *L. nobilis* (26.0 µg·mL^−1^), followed by *Cinnamomum* spp. (21.5–860.0 µg·mL^−1^) and *O. gratissimum* (60.28 to 1053.44 µg·mL^−1^), demonstrating significant activity against flea larvae. In contrast, the highest LC_50_ values, indicating low larvicidal activity, were observed for *Origanum vulgare* (7755.0 µg·mL^−1^), *Mimosa verrucosa* (13,312.5 µg·mL^−1^), and *C. reticulata* (11,530.0 µg·mL^−1^). *Citrus paradisi* showed no detectable biological activity under the evaluated conditions.

The same studies that evaluated larvicidal activity also assessed the activity against pupae. The lowest LC_50_ values were obtained for the EOs of *O. gratissimum* (934.9 µg·mL^−1^), *Cinnamomum* spp. (1730.0 µg·mL^−1^), and *P. cablin* (2305.0 µg·mL^−1^). Conversely, the highest LC_50_ values were recorded for the EOs of *C. sativa* (23,578.0 µg·mL^−1^), *S. sclarea* (23,750.0 µg·mL^−1^), and *L. hybrida* (33,645.0 µg·mL^−1^). The EOs of *C. paradisi* and *M. verrucosa* showed no pupicidal activity at the concentrations and under the method tested.

Six studies evaluated the effect of 12 essential oils on the inhibition of the life cycle of *C. felis*, that is, their ability to interrupt the development from egg to adult. In this context, the lowest LC_50_ values were obtained for the EOs of *S. aromaticum* (15 µg·mL^−1^), *P. aduncum* (25–30 µg·mL^−1^), and *O. gratissimum* (100 µg·mL^−1^). In contrast, the EOs of *L. hybrida* (2390 µg·mL^−1^), *S. sclareae* (3750 µg·mL^−1^), and *C. reticulata* (5000 µg·mL^−1^) had the highest LC_50_ values. Among the species tested, only *C. paradisi* showed no biological activity in relation to life cycle inhibition.

#### 3.2.2. Adult Stages

In the case of adult fleas, 15 published studies were identified, of which 12 and 3 evaluated the activity of essential oils (EOs) against *C. felis* and *X. cheopis*, respectively. For *C. felis*, the activity of 22 EOs was assessed against adult forms (Table 1). The lowest LC_50_ values were obtained for *S. aromaticum* (285.0–829.3 µg·mL^−1^), *O. gratissimum* (292.5–1012.0 µg·mL^−1^), and *P. aduncum* (336–547 µg·mL^−1^). In contrast, the highest LC_50_ values were reported for *M. spicata* (29,878 µg·mL^−1^), *S. sclarea* (44,550 µg·mL^−1^), and *C. sativa* (46,396 µg·mL^−1^). The EO of *C. paradisi* also showed no biological activity against adult *C. felis*.

For *X. cheopis*, the EOs from six plant species were tested against adult fleas (Table 2). The lowest LC_50_ values were observed for *C. verum* (LC_50_ = 0.3 µg·mL^−1^), *Calocedrus decurrens* (LC_50_ = 3.4 µg·mL^−1^), *Salvia rosmarinus* (LC_50_ = 3.7 µg·mL^−1^), *Chamaecyparis lawsoniana* (LC_50_ = 6.11 µg·mL^−1^), *Juniperus occidentalis* (LC_50_ = 11.6 µg·mL^−1^), *Cinnamomum* spp. (LC_50_ = 24.0 µg·mL^−1^), *S. aromaticum* (LC_50_ = 36.3 µg·mL^−1^), *M. piperita* (LC_50_ = 18,000 µg·mL^−1^), and *Eucalyptus globulus* (LC_50_ = 23,700 µg·mL^−1^).

Residual efficacy represents the period during which a treatment remains biologically active after application, reflecting the persistence and stability of the active compounds on the target organism. Among the studies analyzed, three evaluated the residual activity of essential oils from nine plant species against adult *C. felis*. Residual efficacy remained above 90% for the essential oils of *S. sclarea* (2 days), *L. hybrida* (3 days), *Cinnamomum* spp. (4 days), *Pelargonium graveolens* (5 days), *C. reticulata* (6 days), *Origanum vulgare* (6 days), *Thymus vulgaris* (6 days), and *Illicium verum* (8 days).

#### 3.2.3. Overview of Insecticidal Activity of Essential Oil-Based Formulations

Two studies evaluated the development of formulations based on EOs. Nanoemulsions prepared from the EOs of *Cinnamomum* spp. and *S. aromaticum* were tested *in vitro* against adults of *X. cheopis*. The formulations exhibited particle sizes ranging from 80 to 120 nm and showed higher toxicity compared to the pure oils, with reductions in LC_50_ values from 24.01 to 14.50 µg·mL^−1^ for *C. verum* and from 36.26 to 26.42 µg·mL^−1^ for *S. aromaticum*, corresponding to a 27–40% increase in insecticidal activity [122]. Another study evaluated a gel formulation containing 5% EO of *Melaleuca alternifolia* in an *in vivo* assay against *T. penetrans*. The treatment, applied twice daily for three alternating days, resulted in a reduction of more than 70% in parasite viability by the tenth day [123].

**Table 1:** Biological activity of essential oils against different developmental stages of the flea *Ctenocephalides felis felis*.

Essential Oil		LC_50_ (µg·mL^−1^)					Reference
Plant Species	Botanical Family	Eggs	Larvae	Pupae	Adults	Growth-Disrupting Activity	
*Alpinia zerumbet*	Zingiberaceae	653.5	364.5	Not available	27,665.5	Not available	[113]
*Baccharis trimera*	Asteraceae	15,647.2	1747.1	16,385.8	16,206.5	Not available	[124]
*Cannabis sativa*	Cannabaceae	1640.5	4631.2	23,578.5	46,396.0	Not available	[125]
*Cinnamomum* spp.	Lauraceae	90	21.5	Not available	2093.5	Not available	[113]
*Cinnamomum cassia*	Lauraceae	150	860.0	1730.0	3735.0	115.0	[126]
*Citrus paradisi*	Rutaceae	No biological activity	No biological activity	No biological activity	No biological activity	No biological activity	[127]
*Copaifera reticulata*	Fabaceae	No biological activity	11,530.0	11,155.0	23,125.0	5000.0	[127]
*Curcuma zedoaria*	Zingiberaceae	2482.5	3873.0	5154.0	12,775.5	Not available	[128]
*Cymbopogon nardus*	Poaceae	599.0	366.0	Not available	4492.79–29,852.5	Not available	[113]
*Illicium verum*	Schisandraceae	1845.0	720.0	1770.0	6085.0	395.0	[129]
*Laurus nobilis*	Lauraceae	120.5	26.0	Not available	20,604.5	Not available	[113]
*Lavandula hybrida*	Lamiaceae	8570.0	7100.0	33,645.0	27,965.0	2390.0	[127]
*Mentha piperita*	Lamiaceae	Not available	Not available	Not available	8470.8	Not available	[130]
*Mentha spicata*	Lamiaceae	1519.5	628.5	Not available	29,878.0	Not available	[113]
*Mimosa verrucosa*	Fabaceae	9725.0	13,312.5	No biological activity	18,461.0	Not available	[131]
*Ocimum gratissimum*	Lamiaceae	89.5–527.22	60.28–1053.44	934.9	292.50–1012.00	100.0	[113]
*Origanum vulgare*	Lamiaceae	4740.0	7755.0	4920.0	1675.0	310.0	[126]
*Pelargonium graveolens*	Geraniaceae	940.0	1005.0	3380.0	8650.0	625.0	[129]
*Piper aduncum*	Piperaceae	Not available	Not available	Not available	336.0–547.0	25–30	[132]
*Pogostemon cablin*	Lamiaceae	2460.0	3135.0	2305.0	3030.0	Not available	[133]
*Salvia sclarea*	Lamiaceae	19,510.0	5185.0	23,750.0	44,550.0	3750.0	[127]
*Schinus molle* (leaves and fruits)	Anacardiaceae	No biological activity	Not available	Not available	601.0 and 17,697.5	Not available	[107]
*Syzygium aromaticum*	Myrtaceae	Not available	Not available	Not available	285.0–829.3	15.0	[130,134]
*Thymus vulgaris*	Lamiaceae	675.0	1770.0	10,160.0	3225.0	235.0	[126]
*Zanthoxylum limonella*	Rutaceae	Not available	Not available	Not available	9632.0	Not available	[130]
*Zingiber officinale*	Zingiberaceae	Not available	Not available	Not available	11,907.2	Not available	[130]

The authors converted the LC_50_ values to µg·mL^−1^ for standardization purposes.

**Table 2:** Biological activity of essential oils against the adult stages of *Xenopsylla cheopis*.

Essential Oil		LC_50_ (µg·mL^−1^)	Reference
Plant Species	Botanical Family		
*Calocedrus decurrens*	Cupressaceae	3.4	[135]
*Chamaecyparis lawsoniana*	Cupressaceae	6.1	[135]
*Cinnamomum* spp.	Lauraceae	24.0	[122]
*Cinnamomum verum*	Lauraceae	0.3	[136]
*Eucalyptus globulus*	Myrtaceae	23,700	[137]
*Juniperus occidentalis*	Cupressaceae	11.6	[135]
*Mentha piperita*	Lamiaceae	18,000	[137]
*Salvia rosmarinus*	Lamiaceae	3.7	[136]
*Syzygium aromaticum*	Myrtaceae	36.3	[122]

The authors converted the LC_50_ values to µg·mL^−1^ for standardization purposes.

### 3.3. Insecticidal Activity of Bioactive Compounds

**Figure 5:**
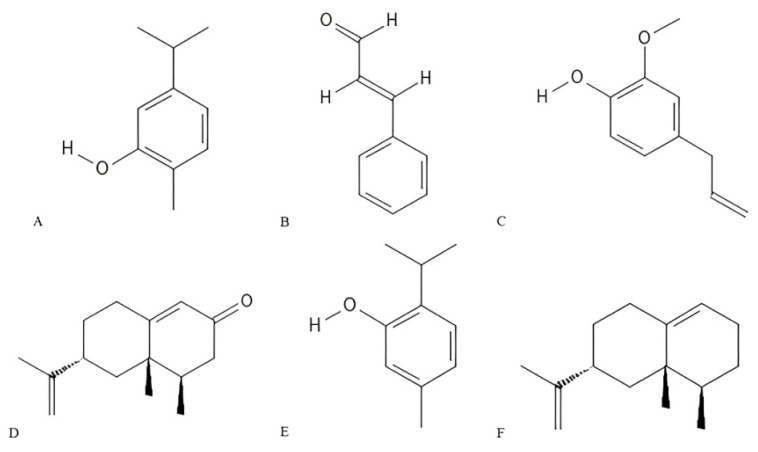
Chemical structures of some major constituents evaluated for flea control activity: carvacrol (**A**); cinnamaldehyde (**B**); eugenol (**C**); nootkatone (**D**); thymol (**E**); and valencene (**F**). Structures were generated using PubChem Sketcher V2.4.

In the reviewed literature, five studies reported *in vitro* insecticidal activity of bioactive compounds against the fleas *C. felis* and *X. cheopis* (Table 3). The chemical structures of the main bioactive compounds with pulicidal activity are illustrated in Figure 5, as generated using PubChem Sketcher V2.4 (https://pubchem.ncbi.nlm.nih.gov/edit3/index.html, accessed on 9 October 2025).

For *C. felis*, only bioactive compounds belonging to the phenylpropanoid class were tested, with eugenol being the only one evaluated against all developmental stages of the flea’s life cycle. Among adults, the lowest LC_50_ value was obtained for eugenol (120.0–259.0 µg·mL^−1^), followed by dillapiole (336.0–547.0 µg·mL^−1^). Regarding life cycle inhibition, the [137] increasing order of toxicity (lowest LC_50_ values) among the tested phenylpropanoids was: eugenol (5.0–6.8 µg·mL^−1^), cinnamaldehyde (8.75 µg·mL^−1^) and dillapiole (25.2–30.3 µg·mL^−1^).

For *X. cheopis*, terpenes (both monoterpenes and sesquiterpenes) were tested. Among the monoterpenes, only carvacrol showed biological activity against this species (LC_50_ = 59.0 µg·mL^−1^). Among the sesquiterpenes, the compounds showing the highest biological activity were nootkatone (LC_50_ = 8.0–18.0 µg·mL^−1^), valencene-13-aldehyde (LC_50_ = 49.0 µg·mL^−1^), and valencene-13-ol (LC_50_ = 83.0 µg·mL^−1^). In contrast, the monoterpenes 3-carene, terpinen-4-ol, and methyl-carvacrol, as well as the sesquiterpenes nootkatin, valencene-11,12-diol, and nootkatone-11,12-epoxide, showed no significant biological activity against adult *X. cheopis*.

#### Overview of Insecticidal Activity in Bioactive-Based Formulations

Two studies evaluated the development of formulations containing bioactive compounds for flea control. The first study developed and evaluated *in vitro* two spray formulations containing eugenol at 1% and 7.5% against adults of *C. felis*. Both formulations showed 100% efficacy after 24 h of exposure and demonstrated residual effects lasting 6 and 48 days, respectively, indicating that increasing the eugenol concentration significantly extended the duration of insecticidal activity [138].

In another study, *in vitro* efficacy was evaluated for both spray and spot-on formulations containing eugenol (10%), carvacrol (10%), and a combination of eugenol + carvacrol (10% + 10%) against adult *C. felis*. For the spray formulations, all caused 100% mortality within 4 h, with the combined formulation showing the fastest knockdown effect, reaching 100% mortality in 2 h. A similar trend was observed for residual efficacy, which remained above 95% for 19 days in formulations containing the individual bioactive compounds and for 24 days in the combined formulation. For the spot-on formulations, all achieved 100% mortality within 45 min, demonstrating a faster knockdown effect compared to the sprays. The combined formulation showed the best performance, reaching 100% mortality in only 15 min. Regarding persistence, the spot-on formulations maintained ≥95% efficacy for up to 10 days, with the eugenol + carvacrol combination again exhibiting the longest residual effect [139].

**Table 3:** Biological activity of bioactive compounds against different developmental stages of the fleas *Ctenocephalides felis felis* and *Xenopsylla cheopis*.

Flea Species	Bioactive Compounds	Chemical Class	Evolutive Forms	LC_50_ (µg·mL^−1^)	Reference
*Ctenocephalides felis*	Eugenol	Phenylpropanoid	Eggs	48.9	[138]
			Larvae	491.8	[138]
			Pupae	58.6	[138]
			Adults	120.0–259.0	[134,138]
			Eggs–Adults	5–6.83	[113,138]
	Dillapiole	Phenylpropanoid	Eggs–Adults	25.2–30.3	[132]
			Adults	336.0–547.0	[132]
	Cinnamaldehyde	Phenylpropanoid	Eggs–Adults	8.8	[124]
*Xenopsylla cheopis*	Carvacrol	Monoterpene	Adults	59.0	[140]
	3-Carene			No biological activity	
	Terpine-4-ol			No biological activity	
	Methyl-carvacrol			No biological activity	
	Nootkatone	Sesquiterpene	Adults	29–83	[140]
	Valencene-13-ol			83.0	
	Valencene-13-aldehyde			85.0	
	Nootkatene			170.0	
	Nootkatone 1–10 epoxide			170.0	
	Nootkatol			240	
	Valencene			410.0	
	Nootkatone--diepoxide			640.0	
	Nootkatin			No biological activity	
	Valencene-11,12-diol			No biological activity	

The authors converted the LC_50_ values to µg·mL^−1^ for standardization purposes.

### 3.4. Repellent Activity of Essential Oils and Bioactive Compounds

When the repellent activity of essential oils against fleas was investigated, four published studies were identified. Two of these studies evaluated, *in vitro*, the activity of 14 essential oils (EOs) and three bioactive compounds against adult *C. felis*. Overall, a wide variation in repellent efficacy was observed among the different EOs tested. Species belonging to the genera *Cinnamomum*, *Copaifera*, *Salvia*, and *Taiwania* showed the best results, with a repellent effect greater than 80%, lasting from 3 to 8 h, depending on the concentration used (Table 4). Among the most promising compounds, the EOs of *C. osmophloeum* and *P. amboinicus* exhibited repellent effects greater than 90%, with persistence of up to 4 to 8 h.

In addition to essential oils, three bioactive compounds showed notable repellent activity against *C. felis*. Cinnamaldehyde (phenylpropanoid) demonstrated a repellent effect greater than 90%, lasting up to 8 h; thymol (monoterpene) exhibited a similar effect for up to 4 h at a concentration of 0.5%; and α-cadinol (sesquiterpene) had a repellent effect greater than 80%, also lasting up to 4 h.

For *P. irritans*, an *in vivo* assay was conducted using the essential oils of four plant species (Table 4). Among the tested oils, *Ziziphora tenuior* exhibited the highest repellent effect (98%), followed by *Myrtus communis* (96%), *Achillea wilhelmsii* (87%), and *Mentha piperita* (69%) after 3 min of exposure.

In an *in vivo* assay, the essential oils of *Mentha pulegium* (Lamiaceae) and *Sassafras albidum* (Lauraceae) demonstrated high repellent efficacy against *D. montanus* (Table 4). Both showed a repellent effect greater than 95% after 20 min of exposure, even at relatively low concentrations, which were 0.41% for *M. pulegium* and 0.14% for *S. albidum.*

## 4. Discussion

This study provides a comprehensive systematic review of the use of essential oils and their bioactive compounds for flea control, consolidating the available knowledge on the topic and highlighting existing gaps in the literature. Although the first report on the application of EOs against fleas dates to 1982, significant progress in this field occurred only after 2011, when a gradual increase was observed in studies evaluating their insecticidal, repellent, and life-cycle inhibitory activities. Up to 2025, a total of 25 articles were identified worldwide, indicating that this remains a scientific field in expansion. In contrast, systematic reviews conducted on other hematophagous arthropods, such as mosquitoes and ticks, reveal a greater number of publications within the same time frame [144,145], suggesting that the use of EOs and their bioactive compounds against fleas remains a promising and still-consolidating area of research.

The species *C. felis* accounts for the largest number of studies involving *in vitro* bioassays with essential oils and bioactive compounds, a result of a combination of biological and practical factors. This flea has a wide geographic distribution and is highly prevalent in cats and dogs, hosts that are easily accessible for collection and experimental maintenance. In contrast, species such as *X. cheopis* and *P. irritans* are difficult to maintain in laboratory colonies, as they do not reproduce successfully for many generations, resulting in occasional and infrequent in viro bioassays [10]. Meanwhile, *T. penetrans* cannot complete its life cycle outside the host, since it depends on penetration into the host’s skin tissue for development [146], so it has been investigated almost exclusively through clinical or *in vivo* observational studies.

Tungiasis is a neglected zoonosis in several countries, and evaluating the efficacy of products through *in vitro* tests poses a major challenge due to the particular biology of this flea species [54,55,56]. In this context, preliminary assays using other flea species may provide valuable information to guide the development of essential oil-based products for its control. These initial investigations can serve as a practical starting point to identify promising formulations and to guide future research toward more effective and sustainable approaches for the management of this parasitosis.

Among the 48 plant species reviewed in this study, 12 belong to the family Lamiaceae. The scientific interest in this family can be attributed to the fact that its members produce essential oils with high chemical diversity and elevated concentrations of monoterpenes and sesquiterpenes, compounds widely recognized for their insecticidal, repellent, and antimicrobial properties [104,112,147]. In addition, this family stands out by including species that are widely cultivated and easy to grow, many of which are traditionally used as aromatic herbs and culinary spices in different regions of the world [148].

Brazil stands out as the country with the largest number of publications evaluating the insecticidal and repellent activity of essential oils and bioactive compounds against fleas, reflecting the growing national interest in the exploration of natural products with veterinary potential. However, among the plant species whose essential oils have been tested, only two, *B. trimera* [131], *M. verrucosa* [131], are native to Brazil, indicating that the country’s vast biodiversity remains largely unexplored from a pulicidal perspective. Since Brazil harbors the greatest botanical diversity on the planet [149,150], this richness represents a strategic reservoir of yet unexplored bioactive compounds, reinforcing the country’s potential for the development of sustainable and innovative alternatives for ectoparasite control.

The results obtained in this review demonstrate the broad spectrum of biological activity of EOs against different life stages of the fleas *C. felis* and adult *X. cheopis*. Due to the difficulty of establishing laboratory colonies of other flea species, the results obtained for immature stages are available only for *C. felis*. For immature stages (egg, larva and pupa), the lowest LC_50_ values were observed for the EOs of *Cinnamomum* spp. [126] and *O. gratissimum* [138], which are rich in the phenylpropanoids cinnamaldehyde and eugenol, respectively. These compounds are known to cause alterations in egg membrane permeability [151,152] and respiratory and cuticular dysfunction in larvae [153] of other arthropod groups.

Regarding life cycle inhibition (from egg to adult), EOs rich in phenylpropanoids, such as those from *Cinnamomum* spp. (LC_50_ = 115 µg·mL^−1^) [126], *S. aromaticum* (LC_50_ = 15 µg·mL^−1^) [134], *P. aduncum* (LC_50_ = 25–30 µg·mL^−1^) [132], and *O. gratissimum* (LC_50_ = 100 µg·mL^−1^) [138] exhibited the lowest LC_50_ values for inhibiting the development from egg to adult in *C. felis*. Even lower doses were recorded for the isolated bioactive compounds eugenol (LC_50_ = 5–6.8 µg·mL^−1^) [134,138], cinnamaldehyde (LC_50_ = 8.75 µg·mL^−1^) [124], and dillapiole (LC_50_ = 25.2–30.3 µg·mL^−1^) [132], whose LC_50_ values were markedly lower than those obtained for the corresponding EOs, demonstrating the high susceptibility of the immature stages to phenylpropanoids. Although some studies have suggested that phenolic compounds may act as IGDs in other arthropods [154,155,156], it remains difficult to determine, based on the methods applied in flea studies, whether the observed effects result from growth interference or from cumulative insecticidal activity across different developmental stages.

For adult stages of *C. felis*, a pattern similar to that observed for immature stages was found, with the lowest LC_50_ values being recorded for the essential oils of *S. aromaticum* [134] (LC_50_ = 285.0–829.3 µg·mL^−1^) [134], *O. gratissimum* (LC_50_ = 292.5–1012.0 µg·mL^−1^) [138], and *P. aduncum* (LC_50_ = 336.0–547.0 µg·mL^−1^) [132,138]. These essential oils are rich in eugenol and dillapiole, both belonging to the phenylpropanoid class. For *C. felis*, only the compounds eugenol (LC_50_ = 120.0–259.0 µg·mL^−1^) [134,138] and dillapiole (LC_50_ = 336.0–547.0 µg·mL^−1^) [132] have been evaluated for their adulticidal efficacy. Several mechanisms have been proposed to explain the toxicity of phenylpropanoids, including inhibition of the insect nervous system, acting on cholinergic, GABAergic, and octopaminergic receptors [154,157], interference with digestive enzymes [158], and inhibition of detoxification-related enzymes [159,160]. However, the exact mechanism responsible for flea mortality remains undefined.

For *X. cheopis*, the lowest LC_50_ values were observed for *C. decurrens* (LC_50_ = 3.4 µg·mL^−1^) [135], *S. rosmarinus* (LC_50_ = 3.7 µg·mL^−1^) [136], *C. lawsoniana* (LC_50_ = 6.11 µg·mL^−1^) [135], and *Juniperus occidentalis* (LC_50_ = 11.6 µg·mL^−1^) [135], showing that EOs rich in oxygenated terpenes exhibit strong adulticidal insecticidal activity against this flea species. Regarding the bioactive compounds tested against adults of *X. cheopis*, a wide variation in biological response was observed. Among the monoterpenes, only carvacrol exhibited pulicidal activity (LC_50_ = 59 µg·mL^−1^) [140], while 3-carene, terpine-4-ol, and methyl-carvacrol showed no insecticidal effect. Among the sesquiterpenes, nootkatone stood out for its higher potency (LC_50_ = 29–83 µg·mL^−1^) [140], followed by valencene-13-ol (83 µg·mL^−1^) [140] and valencene-13-aldehyde (85 µg·mL^−1^) [140].

The differences observed between the two flea species can be attributed to several factors, including morphological and physiological variations, such as differences in the thickness and composition of the cuticle, which influence the penetration and absorption of insecticidal compounds. Another relevant aspect is the differential metabolic capacity, particularly regarding the production, concentration and diversity of detoxifying enzymes such as cytochrome P450, esterases, and glutathione S-transferases, which affect the degradation and efficiency of active compounds [161]. In addition, methodological factors, including the type of treatment applied, exposure time, age or physiological stage of the fleas used, and the temperature at which the tests are maintained, may also have influenced the results obtained [147].

Regarding the repellent activity of essential oils against fleas, studies are still limited. Both *in vitro* and *in vivo* investigations deserve greater attention, given the role of fleas in disease transmission and the importance of bite prevention [3,45,52,53].

The potential of EOs and bioactive compounds against fleas of different species and developmental stages, exhibiting both insecticidal and repellent activities, was demonstrated in this review. Both EOs and bioactive compounds showed clear advantages over synthetic chemicals, including high efficacy, multiple mechanisms of action, low toxicity, and reduced environmental impact^136^. Essential oils may exert synergistic effects due to the combination of their constituents. However, their chemical composition varies according to genotypes and chemotypes, which makes standardization challenging [162]. In contrast, bioactive compounds, often more potent than EOs, have the advantage of being chemically standardized and well characterized.

Most available publications correspond to *in vitro* bioprospecting studies, which represent a valuable source of new candidates for pharmaceutical applications. However, for these compounds to reach the market as safe and effective commercial products, several challenges must still be overcome. The design of pharmaceutical formulations capable of stabilizing and adequately releasing these actives represents one of the major technological challenges, given the inherent instability and volatility of these substances. Essential oils and bioactive compounds are highly susceptible to degradation reactions, such as oxidation, which can lead to reduced or even complete loss of efficacy in formulations [163,164].

In light of these characteristics, the incorporation of EOs and bioactive compounds into nanostructured systems, such as nanoemulsions, nanocapsules, and nanoparticles, is a promising strategy. These systems can enhance the bioactivity of the compounds, since their nanometric size increases tissue penetration and facilitates cellular absorption [163]. Moreover, they allow for controlled release of bioactives, improved dispersibility in aqueous systems, and increased residual efficacy by reducing volatility and protecting the compounds from degradation due to environmental exposure [163,165].

To ensure the safe and rational use of these pharmaceutical compositions in clinical practice, efficacy and safety studies are essential. Most published studies have evaluated only the *in vitro* activity of these products. Therefore, confirming the safety of EOs and their bioactive compounds is crucial for a better understanding of their behavior in mammals. Moreover, important gaps remain regarding the proper registration and regulatory approval of these products for use in flea control. The indiscriminate use of unregistered essential oil-based formulations poses potential risks to animal health, including cases of adverse reactions in dogs and cats exposed to plant-derived flea control products exempt from Environmental Protection Agency regulation in the United States. Despite being marketed as “natural” and “safe”, these products caused clinical signs ranging from mild irritation to severe systemic toxicity, including fatalities. Such findings underscore the importance of toxicological assessments, quality control, and regulatory oversight before these compounds can be safely recommended for clinical or domestic use [166,167].

Overall, EOs and their isolated bioactive compounds represent promising alternatives for flea control due to their broad spectrum of biological activities, rapid environmental degradation, and potential for incorporation into sustainable management strategies. Their main limitations arise from their chemical variability, volatility, and short residual activity, which may reduce consistency in practical applications. These challenges highlight the need for advances in formulation technologies that enhance stability, prolong biological activity, and ensure reproducible effects across different conditions. Future research should also prioritize the evaluation of isolated compounds that have demonstrated superior potency in laboratory assays, as well as the development of standardized preparations that enable reliable *in vivo* testing. Such efforts will be essential to bridge the gap between experimental findings and the development of effective, safe, and technically robust products for flea control.

## 5. Conclusions

In conclusion, EOs and their bioactive compounds exhibit insecticidal and repellent activity against different flea species, with *C. felis* being the most extensively studied. However, the EOs tested so far represent only a small fraction of the vast botanical diversity that remains to be explored. Therefore, further research is needed to identify additional bioactive compounds and to compare their efficacy with that of essential oils. Moreover, new approaches should focus on the standardization of formulations to improve their stability, enabling *in vivo* studies to evaluate the behavior of these compounds in animal models and to confirm their efficacy and safety for use in flea control.

## Figures and Tables

**Table 4:** Repellent activity of essential oils and bioactive compounds against adult fleas of the species *Ctenocephalides felis*, *Diamanus montanus*, and *Pulex irritans*.

Flea Species	Essential Oil/Bioactive Compounds	Botanical Family/Chemical Class	Type of Bioassay	Effective Concentration	Results	Reference
*Ctenocephalides felis*	*Cinnamomum brevipedunculatum*	Lauraceae	*in vitro*	2%	maximum repellent effect of 60% lasting up to 1 h	[141]
	*Cinnamomum osmophloeum*	Lauraceae	*in vitro*	2%	maximum repellent effect of 90% lasting up to 8 h	[141]
	*Citrus paradisi*	Rutaceae	*in vitro*	4%	maximum repellent effect of 60% lasting up to 1 h	[127]
	*Citrus tachibana*	Rutaceae	*in vitro*	2%	maximum repellent effect of 63.5% lasting up to 1 h	[141]
	*Citrus taiwanica*	Rutaceae	*in vitro*	2%	maximum repellent effect of 65.8% lasting up to 1 h	[141]
	*Clausena excavata*	Rutaceae	*in vitro*	2%	maximum repellent effect of 61.4% lasting up to 1 h	[141]
	*Copaifera reticulata*	Fabaceae	*in vitro*	4%	repellent effect greater than 80% lasting up to 3 h.	[127]
	*Cryptomeria japonica*	Cupressaceae	*in vitro*	2%	maximum repellent effect of 55.6% lasting up to 1 h	[141]
	*Cunninghamia konishii*	Cupressaceae	*in vitro*	2%	maximum repellent effect of 58.4% lasting up to 1 h	[141]
	*Gaultheria cumingiana*	Ericaceae	*in vitro*	2%	maximum repellent effect of 54.4% lasting up to 1 h	[141]
	*Lavandula hybrida*	Lamiaceae	*in vitro*	4%	repellent effect greater than 80% lasting up to 1 h	[127]
	*Plectranthus amboinicus*	Lamiaceae	*in vitro*	0.5%	maximum repellent effect of 60% lasting up to 4 h	[141]
	*Salvia sclarea*	Lamiaceae	*in vitro*	4%	repellent effect greater than 80% lasting up to 3 h	[127]
	*Taiwania cryptomerioides*	Cupressaceae	*in vitro*	2%	maximum repellent effect of 80% lasting up to 4 h	[141]
	Cinnamaldehyde	Phenylpropanoid	*in vitro*	2%	repellent effect greater than 90% lasting up to 8 h	[141]
	Thymol	Monoterpene	*in vitro*	0.5%	repellent effect greater than 90% lasting up to 4 h	[141]
	*α-cadinol*	Sesquiterpene	*in vitro*	2%	repellent effect greater than 80% lasting up to 4 h	[141]
*Diamanus montanus*	*Mentha pulegium*	Lamiaceae	*in vivo*	0.41%	repellent effect greater than 95% after 20 min of exposure	[142]
	*Sassafras albidum*	Lauraceae	*in vivo*	0.14%	repellent effect greater than 95% after 20 min of exposure	[142]
*Pulex irritans*	*Achilea wilhelmsii*	Asteraceae	*in vivo*	8%	repellent effect of 87% after 3 min of exposure	[143]
	*Mentha piperita*	Lamiaceae	*in vivo*	8%	repellent effect of 69% after 3 min of exposure	[143]
	*Myrtus communis*	Myrtaceae	*in vivo*	8%	repellent effect of 96% after 3 min of exposure	[143]
	*Ziziphora tenuiore*	Lamiaceae	*in vivo*	8%	repellent effect of 98% after 3 min of exposure	[143]

## Data Availability

No new data were created or analyzed in this study. Data sharing is not applicable to this article.
